# Childhood lymphadenopathy: ultrasonographic predictors of malignancy in a retrospective cohort of 500 patients

**DOI:** 10.3389/fped.2025.1663515

**Published:** 2025-11-06

**Authors:** Şule Çalışkan Kamış, Begül Yağcı

**Affiliations:** Department of Pediatric Hematology and Oncology, Adana Faculty of Medicine, Adana City Education and Research Hospital, University of Health Sciences, Adana, Türkiye

**Keywords:** lymphadenopathy, pediatrics, ultrasonography, biopsy, malignancy, lymphoma

## Abstract

**Introduction:**

Pediatric lymphadenopathy is common and usually benign, yet selecting children who need biopsy remains challenging. We evaluated clinical and ultrasonographic (US) predictors of malignancy in a large pediatric cohort.

**Methods:**

We retrospectively analyzed 500 children (0-18 years) evaluated for regional lymphadenopathy at a tertiary pediatric hematology-oncology clinic (June 2024-March 2025). Demographics, node location/size/count, US features, viral tests, and biopsy/bone-marrow results were extracted. Univariable tests and multivariable logistic regression identified independent predictors of malignancy. Model performance was assessed with Hosmer-Lemeshow and ROC AUC.

**Results:**

Median age was 6 years; cervical nodes predominated (92.2%), and multiple nodes were frequent (93.6%). Biopsy was performed in 47 children; malignancy was found in 23. Final diagnoses in the cohort were reactive (87.4%), infectious (6.8%), hematologic malignancy (4.4%), and solid tumor metastasis (1.4%). On univariable analysis, node size ≥2 cm, multiplicity, and region were associated with malignancy (*p* < 0.05). In multivariable analysis, only US assessment remained independently predictive: nodes categorized as suspicious on US had ∼56-fold higher odds of malignancy (adjusted OR ≈ 55.6; 95% CI 14.3-200; *p* < 0.001), whereas size and region were not significant. The model showed good calibration and excellent discrimination (AUC = 0.92; 95% CI 0.88-0.96).

**Discussion:**

US features outperformed traditional parameters (size/site) for predicting malignancy. Incorporating US-driven criteria into pathways may reduce unnecessary biopsies while preserving timely cancer detection.

## Introduction

Childhood regional lymphadenopathy (LAP) is a common clinical finding and is most often related to benign causes such as acute upper respiratory tract infections or reactive hyperplasia. Epidemiological studies suggest that up to 40% of healthy children may experience lymph node enlargement at some point during childhood (1–3). Infections, particularly viral, are the predominant etiology, typically associated with nonspecific follicular and paracortical hyperplasia (4, 5).

Among anatomical regions, cervical lymph nodes are most frequently affected. While most cases are self-limiting, persistent lymphadenopathy can indicate serious conditions, including suppurative or granulomatous lymphadenitis, hematologic malignancies, or rarer disorders such as Langerhans cell histiocytosis, Rosai–Dorfman disease, and Castleman disease (6). In addition, inborn errors of immunity (IEI) and immune dysregulation disorders may also present with recurrent or persistent lymphadenopathy, reflecting underlying defects in immune regulation and predisposing affected children to infections or lymphoproliferative disease (7). Distinguishing benign from malignant causes is often challenging; although biopsy may be necessary, its invasive nature mandates careful selection (8).

Reliable clinical and radiological predictors are therefore needed to guide biopsy decisions. Ultrasonography (US), in particular, has emerged as a non-invasive tool that provides valuable diagnostic information. We aimed to determine the etiologies of childhood regional lymphadenopathy and to analyze risk factors—especially ultrasonographic features—associated with malignancy in a large pediatric cohort.

## Materials and methods

This retrospective study included 500 pediatric patients aged 0–18 years who were evaluated with the diagnosis of regional lymphadenopathy between June 1, 2024, and March 1, 2025, at the Pediatric Hematology and Oncology Clinic of Adana City Training and Research Hospital, affiliated with the Health Sciences University, Adana Faculty of Medicine. All consecutive patients presenting to or followed up at our clinic during this time period with a diagnosis of regional lymphadenopathy were included. Patients with incomplete demographic, laboratory, or imaging data were excluded. Patient data were retrospectively retrieved from the hospital's automation system and anonymized before analysis.

A structured data collection form was used, which included demographic information, the anatomical location of the enlarged lymph node, ultrasonography (US) findings, viral screening results, lymph node biopsy, and bone marrow examination results. The following data were collected for each patient: sex, birth date, age, region of lymphadenopathy (cervical, axillary, mediastinal, abdominal, inguinal**),** and US findings (number of lymph nodes, size, presence of hepatosplenomegaly, and LAP assessment). Viral tests for Epstein–Barr virus (EBV), cytomegalovirus (CMV), parvovirus, and TORCH infections were recorded as either positive or negative. Clinical features such as consistency, mobility, tenderness, and systemic symptoms (fever, weight loss, night sweats) were not systematically documented in hospital records and therefore could not be included in the analysis.

### Ultrasonography protocol and image acquisition

All ultrasound examinations were performed by board-certified pediatric radiologists using high-frequency linear transducers (7–15 MHz) on contemporary scanners. Standardized planes (longitudinal and transverse) were obtained. For color Doppler, the pulse repetition frequency was set at a low range (≈0.8–1.2 kHz) with a low wall filter to detect slow flow, and gain was optimized to avoid blooming artifacts. The following features were prospectively or retrospectively recorded: nodal size (short and long axes), short-to-long axis ratio, cortical thickness (pathological if >3 mm), echotexture (homogeneous vs. heterogeneous), margin regularity, presence or absence of an echogenic fatty hilum, and vascular pattern (hilar vs. peripheral/mixed). Nodes were categorized as reactive, suspicious, or malignancy-suspected based on the pre-specified criteria detailed above. Images were reviewed by two pediatric radiologists in consensus, and any disagreements were resolved by joint reading. Elastography was not available during the study period and therefore was not performed.

### Ultrasonography assessment

Classification as suspicious or malignancy-suspected on ultrasonography was based on the presence of one or more abnormal features, including loss of the fatty hilum, rounded shape (short-to-long axis ratio >0.5), irregular margins, heterogeneous echotexture, cortical thickening (>3 mm), and/or abnormal hilar or peripheral vascularity on Doppler.

### Biopsy indications

Biopsy was considered for persistent nodes (>6–8 weeks), mediastinal location, supraclavicular location (if available), B symptoms (fever, weight loss, night sweats), rapidly enlarging nodes, or suspicious US features as defined above.

Lymph node biopsy was performed when indicated, and the pathological results were categorized as reactive, granulomatous, or malignant. Additionally, bone marrow flow cytometry results were reviewed. The final diagnosis was determined as reactive lymphadenopathy, infectious lymphadenopathy, hematological malignancy (leukemia/lymphoma), or solid tumor metastasis. Follow-up duration was recorded as 0–6 months, 6–12 months, or over 12 months, and the patient's final status was classified as follow-up, complete recovery, or disease progression.

**Figure 1:**
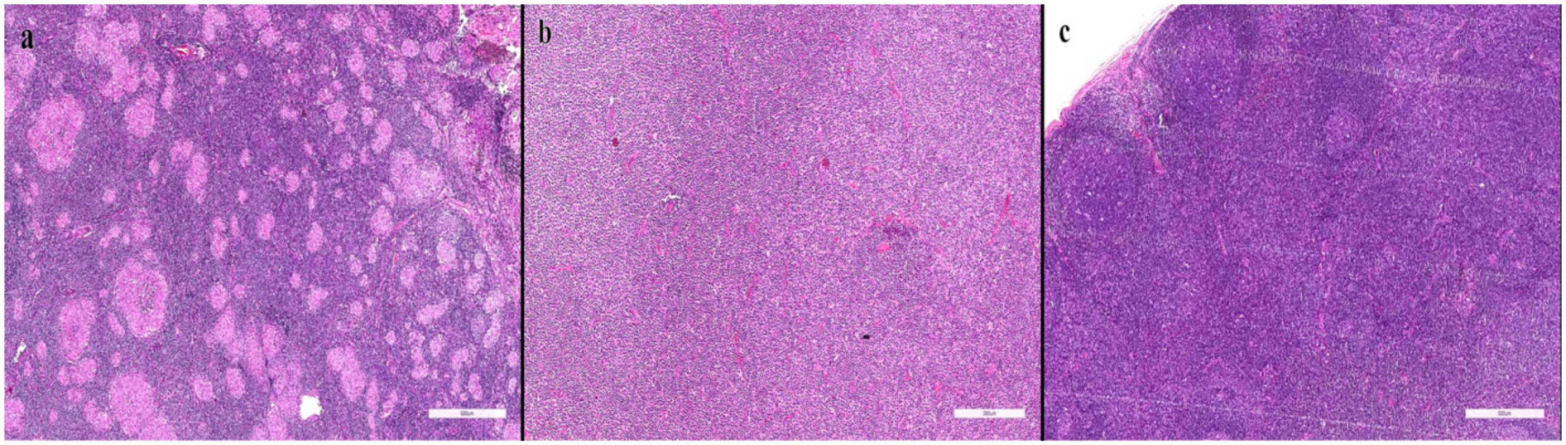
Representative histopathology (reactive, granulomatous, malignant). **(a)** Granulomatous lymphadenitis characterized by epithelioid histiocytes and multinucleated giant cells. **(b)** Diffuse large B-cell lymphoma demonstrating effacement of normal lymph node architecture by sheets of atypical large lymphoid cells. **(c)** Reactive lymphoid hyperplasia showing preserved architecture with prominent germinal centers.

Numbers of procedures were specified as follows: fine-needle aspiration cytology (FNAC) was performed in 12 cases, excisional/incisional histopathology in 47 cases, and immunohistochemistry (IHC) in 28 cases; immunocytochemistry was not performed. Representative histopathology images are presented in Figure 1.

The relationship between lymph node size, the presence of multiple lymph nodes, lymph node location, and the final diagnosis was analyzed using statistical methods. Parametric and non-parametric tests were applied where appropriate. Data analysis was conducted using SPSS version 26.0 (IBM Corp., Armonk, NY, USA) or similar statistical software.

### Statistical analysis

Categorical variables were presented as counts and percentages, while continuous variables were expressed as mean ± standard deviation or median (range), depending on distribution. The normality of distribution was assessed using the Kolmogorov–Smirnov test. For comparisons, the Student's *t*-test was applied to normally distributed continuous variables, and the Mann–Whitney *U* test was used for non-normally distributed variables. The chi-square test was used to compare categorical variables, and the Kruskal–Wallis test was employed for comparisons among three or more independent groups when data were not normally distributed. Correlation analysis was conducted using Pearson's test for normally distributed variables and Spearman's test for non-normally distributed variables.

The statistical significance level was set at two-sided *p* ≤ 0.05. In addition, multivariable binary logistic regression analysis was performed to determine independent predictors of malignancy, and results were reported as adjusted odds ratios (OR) with 95% confidence intervals (CI). Where applicable, relative risks (RR) with 95% CI were calculated for two-by-two comparisons. Model calibration and discrimination were evaluated using the Hosmer–Lemeshow goodness-of-fit test and the area under the receiver operating characteristic (ROC) curve, with the area under the curve (AUC) reported.

### Coding and reference categories

The binary outcome (malignancy) was coded 1 = present and 0 = absent; reference categories were reactive US (US groups), cervical region (location), size <2 cm (size), and single node (count).

The *a priori* sample size calculation indicated that approximately 288 patients in total (144 in each comparison group) would have been sufficient to detect the observed difference in malignancy rates with 80% power at a significance level of *α* = 0.05. However, due to the retrospective design of the study, all eligible patients during the study period (*n* = 500) were included, thereby further enhancing statistical power. This ensured that the study was adequately powered for detecting the observed associations.

## Results

Our study included a total of 500 patients. Of these, 32.6% were female (*n* = 163) and 67.4% were male (*n* = 337), with a higher proportion of male patients. The median age was 6 years (range: 0–18). The most frequently affected region was the cervical lymph nodes, with enlargement detected in 92.2% (*n* = 461). Enlargement was observed in the axillary lymph nodes in 1.4% (*n* = 7), mediastinal in 0.4% (*n* = 2), abdominal in 3.2% (*n* = 16), and inguinal in 2.8% (*n* = 14).

Lymphadenopathy predominantly involved multiple lymph nodes (93.6%, *n* = 468), while a single lymph node was observed in 6.4% (*n* = 32). Lymph node size was <2 cm in 43.4% (*n* = 217) and ≥2 cm in 56.6% (*n* = 283). Hepatosplenomegaly (HSM) was present in 10.2% (*n* = 51).

According to ultrasonographic (US) examinations, reactive lymphadenopathy was detected in 457 patients (91.4%), suspicious in 30 (6.0%), and malignancy-suspected in 13 (2.6%). Among the 47 biopsied patients, fine-needle aspiration cytology (FNAC) was performed in 12, histopathology in all 47, and immunohistochemistry (IHC) in 28. Representative micrographs are provided (Figure 1).

Viral test results were positive in 37 patients (7.4%), of which Epstein–Barr virus (EBV) accounted for 83.8%, cytomegalovirus (CMV) for 13.5%, and TORCH infection for 2.7% (Table 1).

**Table 1:** Distribution of viral test results (positive cases).

Viral Test Result	Frequency	Percent (%)	Valid Percent (%)
EBV	31	6.2	83.8
CMV	5	1	13.5
TORCH	1	0.2	2.7
Total	37	7.4	100

Percent = *n*/500 × 100; Valid Percent = *n*/37 × 100.

TORCH (Toxoplasmosis, Other infections, Rubella, Cytomegalovirus, Herpes simplex).

Lymph node biopsy was performed in 47 patients (9.4%). Pathology revealed reactive lymphadenopathy in 20 (42.6%), granulomatous in 4 (8.5%), and malignant in 23 (48.9%) (Table 2).

**Table 2:** Lymph node pathology results.

Pathology Result	Frequency	Percent (%)	Valid percent (%)
Reactive	20	4	42.6
Granulomatous	4	0.8	8.5
Malignant	23	4.6	48.9
Total	47	9.4	100

Percent = *n*/500 × 100; Valid Percent = *n*/47 × 100.

**Figure 2:**
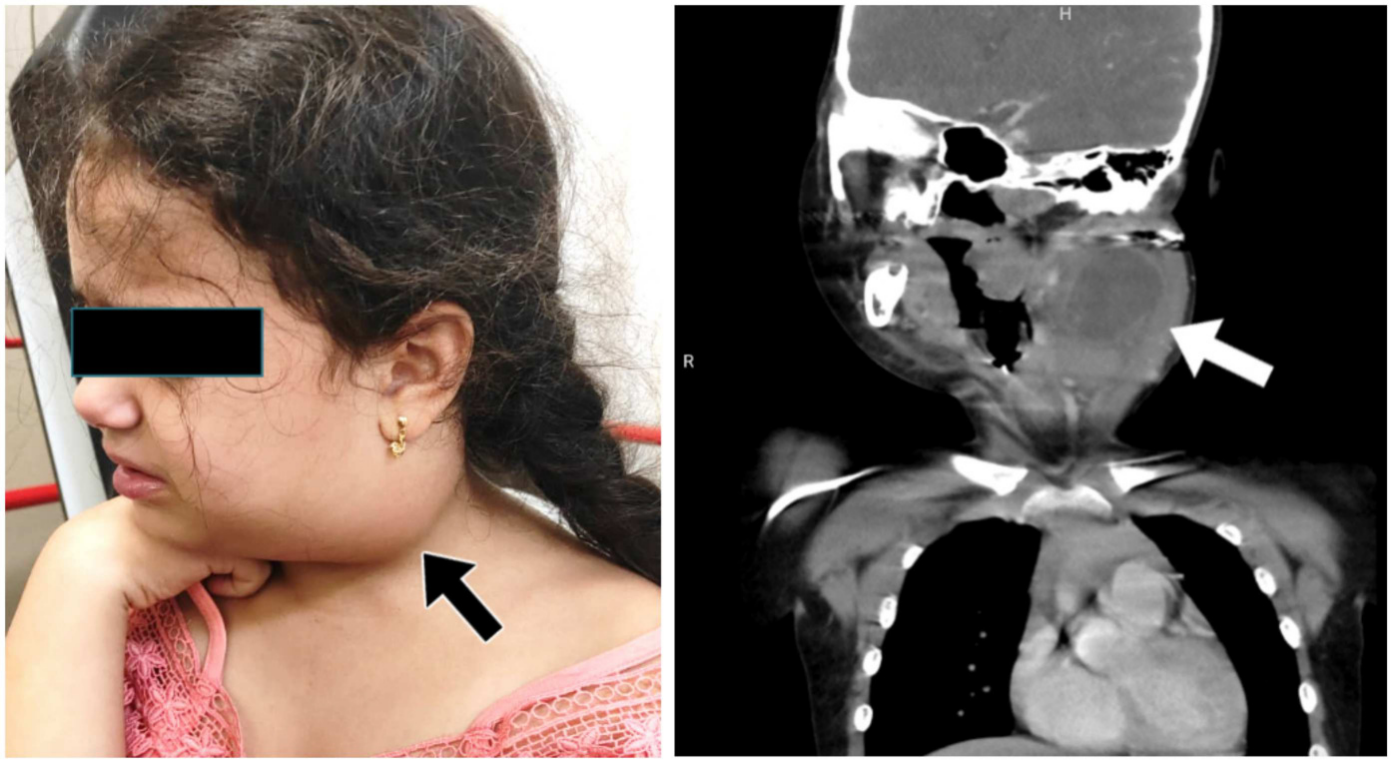
Representative clinical and radiological findings. **(a)** Clinical photograph demonstrating a visibly enlarged lymph node in the left cervical region (arrow). The node was non-tender, rubbery in consistency, and measured approximately 3 cm, rapidly growing over two weeks. **(b)** Contrast-enhanced neck CT showing multiple enlarged cervical lymph nodes, the largest measuring 29 mm × 41 mm in the left submandibular posterior region (arrow), with central necrosis suggestive of pathological lymphadenopathy. This case is presented as a representative illustration and not as a determinant of the statistical results.

Representative clinical and radiological findings are presented in Figure 2. The clinical photograph (Figure 2a) shows a visibly enlarged lymph node in the left cervical region, which had rapidly increased in size within a short period. On physical examination, the node was non-tender, rubbery in consistency, and measured approximately 3 cm, prompting further evaluation. The contrast-enhanced neck CT (Figure 2b) revealed multiple enlarged cervical lymph nodes, the largest measuring 29 mm × 41 mm in the left submandibular posterior region, with central necrosis. Such radiological features are frequently associated with pathological lymphadenopathy and raise suspicion for malignancy. Subsequently, whole-body FDG PET-CT demonstrated intense uptake in a 40 mm × 30 mm tonsillar mass extending into and narrowing the pharyngeal lumen (SUVmax: 16), along with mild uptake in bilateral cervical lymph nodes (SUVmax: 2.3). No pathological FDG uptake was detected in the thorax, abdomen, pelvis, or skeletal system. Excisional biopsy of the left level II cervical lymph node showed effacement of nodal architecture by diffuse infiltration of large atypical lymphoid cells. Immunohistochemistry was positive for CD20, CD5, CD79a, MUM1, LCA, Bcl-2, and Bcl-6, while negative for TdT, CD10, synaptophysin, desmin, CD3, and CD88. The Ki-67 proliferation index was approximately 90%. These findings were consistent with a diagnosis of diffuse large B-cell lymphoma, non-germinal center B-cell (non-GCB) subtype.

Final diagnoses in the entire cohort were: reactive lymphadenopathy in 437 (87.4%), infectious lymphadenopathy in 34 (6.8%), hematologic malignancy in 22 (4.4%), and metastatic solid tumors in 7 (1.4%) (Table 3).

**Table 3:** Final diagnosis distribution.

Diagnosis	Frequency	Percent (%)
Reactive Lymphadenopathy	437	87.4
Infectious Lymphadenopathy	34	6.8
Hematologic Malignancy (Leukemia/Lymphoma)	22	4.4
Solid Tumor Metastasis	7	1.4
Total	500	100

According to follow-up duration, 32 patients (6.4%) were followed for 0–6 months, 57 (11.4%) for 6–12 months, and 411 (82.2%) for >12 months. Disease progression occurred in 26 patients (5.2%). Importantly, no patient initially managed as reactive or infectious lymphadenopathy without biopsy subsequently developed malignancy during follow-up (most >12 months).

### Multivariable logistic regression

**Figure 3:**
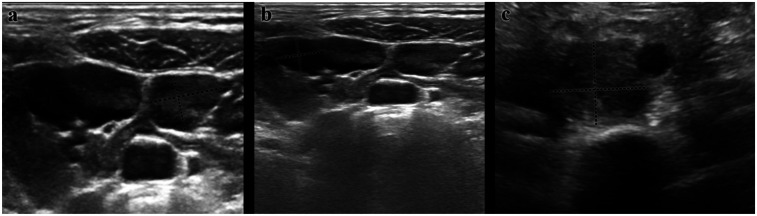
Representative ultrasonographic images of cervical lymph nodes. **(a)** Reactive LAP: Oval shape, preserved echogenic hilum, thin cortex, and regular margins; Doppler shows hilar vascularity. **(b)** Suspicious LAP: Rounded morphology, cortical thickness >3 mm, heterogeneous echotexture, and mixed hilar/peripheral vascularity. **(c)** High suspicion of malignancy LAP: Loss of hilum, irregular margins, marked cortical thickening, and predominant peripheral vascularity.

In the multivariable logistic regression analysis, ultrasonographic findings were the only independent predictor of malignancy. Nodes categorized as suspicious on ultrasound were associated with ∼56-fold higher odds of malignancy (adjusted OR ≈ 55.6; 95% CI: 14.3–200; *p* < 0.001), whereas lymph node size and anatomical region did not retain statistical significance (*p* > 0.05). Representative ultrasonographic appearances of reactive, suspicious, and high-suspicion nodes are shown (Figure 3). The overall model was statistically significant (Omnibus *χ*^2^ = 133.9, *df* = 4, *p* < 0.001), explained ∼67% of the variance (Nagelkerke *R*^2^ = 0.670), and showed good calibration (Hosmer–Lemeshow *χ*^2^ = 1.96, *df* = 2, *p* = 0.376). Classification accuracy was 97.4%, with very high specificity (99.6%) but limited sensitivity (60.7%) (Table 4). Estimates for the “malignancy-suspected” stratum were unstable due to sparse data (quasi-complete separation). Additional binary analysis combining suspicious and malignancy-suspected categories is provided in Supplementary Table 1, confirming the robustness of the findings.

**Table 4:** Multivariable logistic regression analysis of predictors of malignancy.

Variable	*B*	SE	Wald	*p*-value	Adjusted OR (Exp(*B*))	95% CI for OR
Enlarged lymph node region	−0.359	0.191	3.524	0.060	0.699	0.480–1.016
Lymph node size ≥2 cm	−0.178	0.770	0.053	0.818	0.837	0.185–3.787
US (suspicious vs. reactive)	−4.003	0.687	33.985	<0.001	0.018	0.005–0.070
US (Malignancy-suspected vs. reactive)	−25.814	11,004.280	0.000	0.998	0.000	—
Constant	5.542	1.413	15.380	<0.001	255.179	—

Model fit statistics: Omnibus *χ*^2^ = 133.923 (*df* = 4), *p* < 0.001; Nagelkerke *R*^2^ = 0.670; Hosmer–Lemeshow *χ*^2^ = 1.957 (*df* = 2), *p* = 0.376; overall accuracy = 97.4% (specificity = 99.6%, sensitivity = 60.7%).

For interpretability, the reciprocal of the OR for US (Suspicious vs. Reactive) can be reported as ≈55.6 (95% CI: ≈14.3–200).

### Association between enlarged lymph node region and final diagnosis

Reactive lymphadenopathy was most frequently observed in the cervical region (93.6%, *n* = 409). Fewer cases were detected in the axillary (1.4%, *n* = 6), abdominal (2.5%, *n* = 11), and inguinal (2.5%, *n* = 11) regions; none were identified in the mediastinal region. Infectious lymphadenopathy was also predominantly cervical (91.2%, *n* = 31). Hematologic malignancies were most commonly cervical (81.8%, *n* = 18), with additional cases in abdominal (9.1%, *n* = 2) and mediastinal (4.5%, *n* = 1) regions. Solid tumor metastases were identified in cervical (42.9%, *n* = 3) and abdominal (42.9%, *n* = 3) regions, with one mediastinal case (14.3%). Overall differences by region were significant (*p* < 0.001) (Table 5).

**Table 5:** Association between enlarged lymph node region and final diagnosis.

Enlarged lymph node region	Reactive lymphadenopathy	Infectious lymphadenopathy	Hematological malignancy (leukemia/lymphoma)	Solid tumor metastasis	Total
Cervical	409 (93.6%)	31 (91.2%)	18 (81.8%)	3 (42.9%)	461 (92.2%)
Axillary	6 (1.4%)	1 (2.9%)	0 (0%)	0 (0%)	7 (1.4%)
Mediastinal	0 (0%)	0 (0%)	1 (4.5%)	1 (14.3%)	2 (0.4%)
Abdominal	11 (2.5%)	0 (0%)	2 (9.1%)	3 (42.9%)	16 (3.2%)
Inguinal	11 (2.5%)	2 (5.9%)	1 (4.5%)	0 (0%)	14 (2.8%)
Total	437 (100%)	34 (100%)	22 (100%)	7 (100%)	500 (100%)

### Association between lymph node size and final diagnosis

Reactive lymphadenopathy occurred more often with nodes <2 cm (46.9%, *n* = 205), though 53.1% (*n* = 232) of reactive cases had nodes ≥2 cm. Infectious lymphadenopathy clustered in the ≥2 cm group (76.5%, *n* = 26). Hematologic malignancies were strongly linked to ≥2 cm nodes (86.4%, *n* = 19), as were solid tumor metastases (85.7%, *n* = 6). The association between size category and final diagnosis was significant (*p* < 0.001) (Table 6).

**Table 6:** Association between lymph node size and final diagnosis.

Lymph node size	Reactive lymphadenopathy	Infectious lymphadenopathy	Hematological malignancy (leukemia/lymphoma)	Solid tumor metastasis	Total
<2 cm	205 (46.9%)	8 (23.5%)	3 (13.6%)	1 (14.3%)	217 (43.4%)
≥2 cm	232 (53.1%)	26 (76.5%)	19 (86.4%)	6 (85.7%)	283 (56.6%)
Total	437 (100%)	34 (100%)	22 (100%)	7 (100%)	500 (100%)

### Association between lymph node count and final diagnosis

There was a significant difference between single and multiple node involvement (*p* = 0.001). Multiple nodes predominated among hematologic malignancies (95.5%, 21/22) and infectious lymphadenopathy (94.1%, 32/34), whereas solitary nodes were relatively more frequent in solid tumor metastasis (42.9%, 3/7) (Table 7).

**Table 7:** Distribution of lymph node count by final diagnosis.

Lymph node count	Reactive lymphadenopathy	Infectious lymphadenopathy	Hematological malignancy (leukemia/lymphoma)	Solid tumor metastasis	Total
Single	26 (5.9%)	2 (5.9%)	1 (4.5%)	3 (42.9%)	32 (6.4%)
Multiple	411 (94.1%)	32 (94.1%)	21 (95.5%)	4 (57.1%)	468 (93.6%)
Total	437 (100%)	34 (100%)	22 (100%)	7 (100%)	500 (100%)

### Association between biopsy status and final diagnosis

Final diagnoses differed by biopsy status (*p* < 0.001). Biopsy was performed in 47 patients (9.4%). Reactive lymphadenopathy was predominantly diagnosed without biopsy (96.6%, 422/437), as was infectious lymphadenopathy (82.4%, 28/34). All hematologic malignancies underwent biopsy (100%, 22/22). Among solid tumor metastases, 57.1% (4/7) had biopsy (Table 8).

**Table 8:** Distribution of lymph node biopsy status by final diagnosis.

Lymph node biopsy status	Reactive lymphadenopathy	Infectious lymphadenopathy	Hematological malignancy (leukemia/lymphoma)	Solid tumor metastasis	Total
Yes	15 (3.4%)	6 (17.6%)	22 (100%)	4 (57.1%)	47 (9.4%)
No	422 (96.6%)	28 (82.4%)	0 (0.0%)	3 (42.9%)	453 (90.6%)
Total	437 (100%)	34 (100%)	22 (100%)	7 (100%)	500 (100%)

### Determination of lymphadenopathy type

Pre-biopsy classification integrated clinical, US, and laboratory findings into three categories: reactive, suspicious, and malignancy-suspected. Reactive nodes typically showed smooth borders, homogeneous echotexture, preserved fatty hilum, and normal Doppler vascularity. Suspicious nodes exhibited one or more concerning features (rounded shape, irregular margins, heterogeneity, cortical thickening >3 mm, abnormal vascularity). Malignancy-suspected nodes combined multiple abnormal US features and/or clinical red flags (rapid growth, B symptoms) and were prioritized for histopathology.

### Association between lymphadenopathy type and final diagnosis

Definitive diagnoses differed significantly across categories (*p* < 0.001). The reactive category was largely benign (reactive 93.0%; infectious 6.1%; hematologic malignancy 0.7%). The suspicious group showed higher proportions of infectious (1.3%) and hematologic malignancy (1.8%), with some solid metastasis (0.9%). The malignancy-suspected group was predominantly malignant (hematologic 2.4%; solid metastasis 0.4%) (Table 9).

**Table 9:** Distribution of definitive diagnosis by lymphadenopathy type.

Lymphadenopathy type	Reactive lymphadenopathy	Infectious lymphadenopathy	Hematological malignancy (leukemia/lymphoma)	Solid tumor metastasis	Total
Reactive	425 (93.0%)	28 (6.1%)	3 (0.7%)	1 (0.2%)	457 (91.4%)
Suspicious	12 (2.7%)	6 (1.3%)	8 (1.8%)	4 (0.9%)	30 (6.0%)
Malignancy suspected	0 (0.0%)	0 (0.0%)	11 (2.4%)	2 (0.4%)	13 (2.6%)
Total	437 (100%)	34 (100%)	22 (100%)	7 (100%)	500 (100%)

Values are presented as *n* (%).

In the multivariable logistic regression analysis, ultrasonographic findings emerged as the only independent predictor of malignancy. Nodes classified as suspicious on ultrasound were associated with approximately 56-fold higher odds of malignancy (adjusted OR ≈ 55.6; 95% CI: 14.3–200; *p* < 0.001), whereas lymph node size and anatomical region did not retain statistical significance (*p* > 0.05). The model was statistically significant (Omnibus *χ*^2^ = 133.9, *df* = 4, *p* < 0.001), explained ∼67% of the variance in malignancy (Nagelkerke *R*^2^ = 0.670), and showed good calibration (Hosmer–Lemeshow *χ*^2^ = 1.96, *df* = 2, *p* = 0.376). The overall classification accuracy was 97.4%, with very high specificity (99.6%) but limited sensitivity (60.7%) (Table 4).

**Figure 4:**
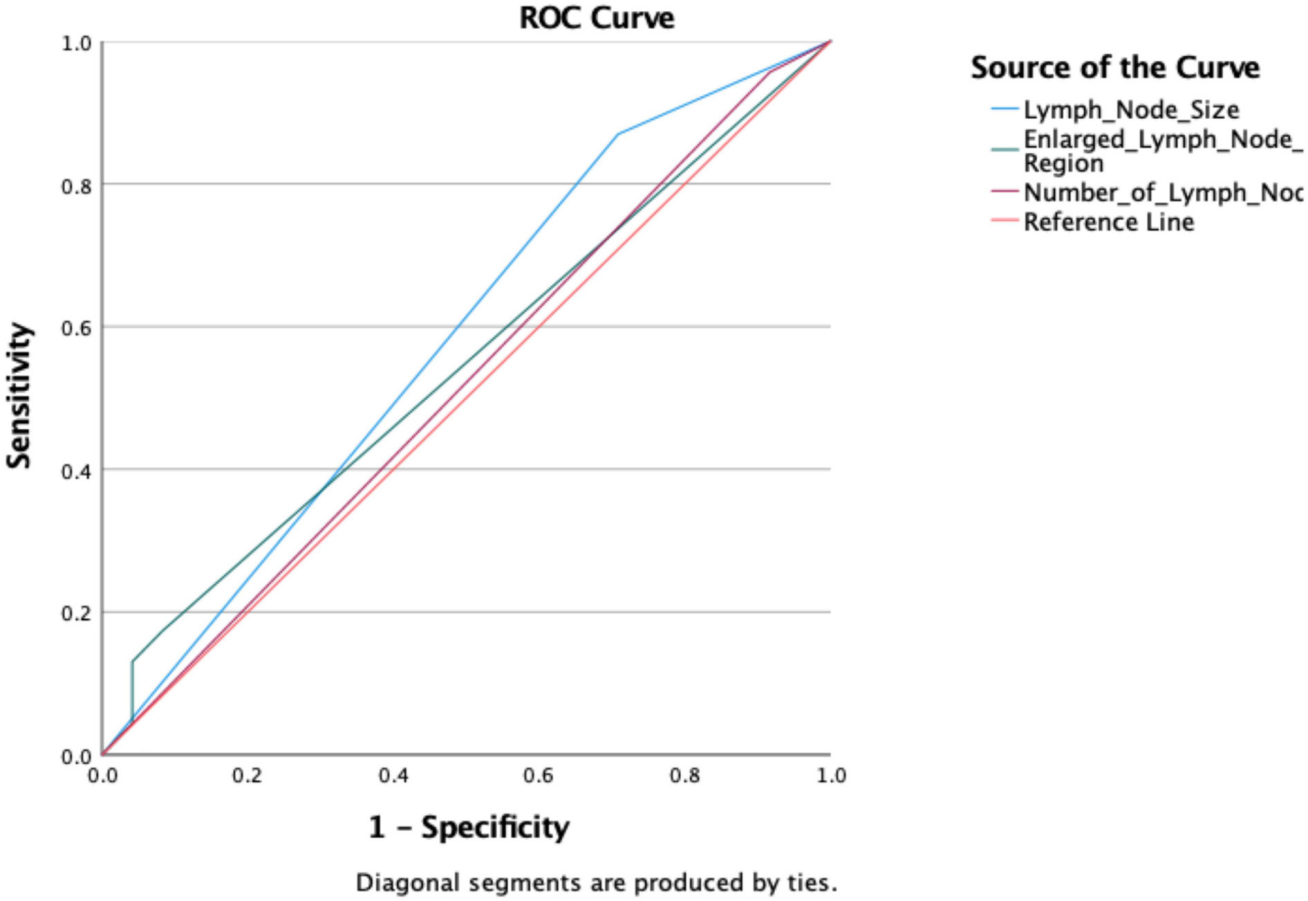
Receiver operating characteristic (ROC) curves for lymph node size, enlarged lymph node region, and lymph node count, showing limited discrimination for malignancy (AUC = 0.581, 0.545, and 0.520, respectively).

**Figure 5:**
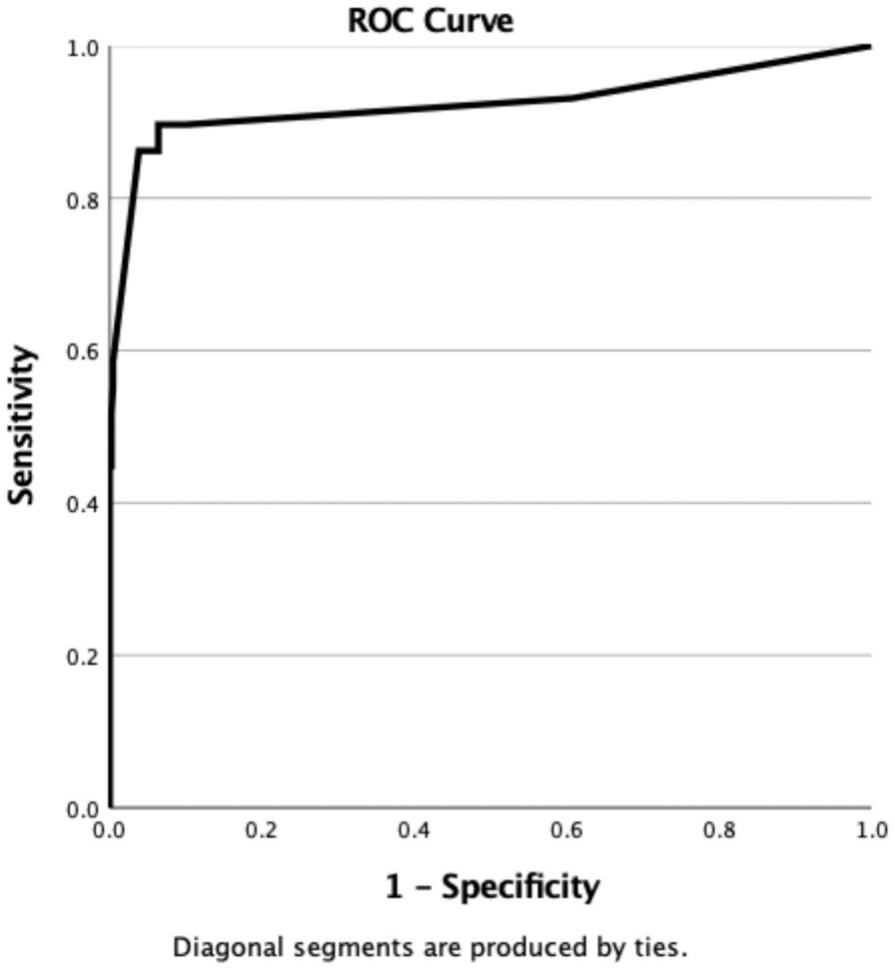
ROC curve of the multivariable logistic regression model predicting malignancy in childhood lymphadenopathy (AUC = 0.92; 95% CI: 0.88–0.96).

We then evaluated model discrimination. ROC analysis demonstrated that lymph node size, enlarged lymph node region, and number of lymph nodes had limited discriminatory power for malignancy (AUC = 0.581, 0.545, and 0.520, respectively) (Figure 4). In contrast, the multivariable logistic regression model achieved excellent discrimination, with an AUC of 0.92 (95% CI: 0.88–0.96), indicating strong ability to distinguish malignant from benign lymphadenopathy (Figure 5).

## Discussion

In this large retrospective cohort of 500 children with lymphadenopathy, the cervical region was the most frequently affected site, and reactive lymphadenopathy was the most common diagnosis (9). The overall malignancy rate was 5.8%, which is consistent with previously reported rates of 4%–6% in pediatric cohorts undergoing further evaluation (10, 11). These findings confirm that while lymphadenopathy in children is usually benign, a significant minority of cases are malignant and warrant careful diagnostic assessment.

Univariate analyses revealed that lymph node size ≥2 cm, multiplicity of lymph nodes, and supraclavicular or mediastinal localization were significantly associated with malignancy. These results reinforce classical pediatric evaluation principles that larger nodes, multiple sites, and certain anatomical localizations are red flags for malignancy.

However, in multivariable logistic regression, only ultrasonographic findings remained an independent predictor of malignancy. Nodes classified as suspicious or malignancy-suspected on ultrasound showed markedly increased odds of malignancy, with suspicious nodes demonstrating an approximately 56-fold higher risk (adjusted OR ≈ 55.6, 95% CI ≈ 14.3–200, *p* < 0.001), while node size, multiplicity, and anatomical region lost significance after adjustment. This underscores the central role of ultrasonography compared to traditional clinical parameters.

Consistent with the cohort-level findings, the illustrative case in Figure 2 underscores that imaging-driven suspicion—operationalized in this study via ultrasonographic criteria—rather than size or site alone, aligns with the subsequent malignant diagnosis, thereby paralleling our multivariable results.

These findings align with recent studies reporting that specific US features—loss of fatty hilum, irregular margins, heterogeneous echotexture, and abnormal vascularity—are superior predictors of malignancy compared with size or site alone (1, 5, 8). Our results therefore support an ultrasound-centered approach to biopsy decision-making in pediatric lymphadenopathy.

Recent advances, particularly shear-wave and strain elastography, have been explored to improve risk stratification of pediatric lymph nodes by quantifying stiffness (12, 13). Although elastography was not available in our center during the study period, incorporating elastographic parameters into standardized US assessment may further enhance discrimination between benign and malignant nodes. Prospective multicenter studies that integrate gray-scale, Doppler, and elastography with uniform thresholds are warranted (12, 13).

The malignancy yield among biopsied patients in our study was 48.9%, reflecting stringent biopsy selection in a tertiary referral center. While this high diagnostic yield strengthens the clinical value of our findings, it also highlights the presence of selection and referral bias—an inherent limitation of retrospective single-center studies.

Representative histopathological images were included to illustrate the spectrum of reactive, granulomatous, and malignant changes, addressing the reviewer's request for microscopic documentation. This provides additional clarity regarding the pathological spectrum observed in pediatric lymphadenopathy.

Our *post hoc* power analysis confirmed that the study was adequately powered to detect the effects of ultrasound findings and lymph node size, with power levels exceeding 95%. Conversely, the comparison of single vs. multiple nodes demonstrated low statistical power due to the small number of solitary-node patients, and this result should therefore be interpreted cautiously. In addition, *a priori* sample size calculation indicated that approximately 288 patients (144 in each comparison group) would have been sufficient for 80% power, and our actual sample (*n* = 500) exceeded this threshold, further increasing reliability (2, 4, 11).

Taken together, these findings demonstrate that while node size and location provide useful clinical clues, ultrasonography is the most reliable independent predictor of malignancy in childhood lymphadenopathy. Future multicenter, prospective studies with standardized ultrasonographic protocols are warranted to validate these predictors and to establish evidence-based diagnostic algorithms (1, 5, 8).

### Clinical implications and practical diagnostic approach

Our findings emphasize the central role of ultrasonography in evaluating childhood lymphadenopathy. Based on these results, we propose a preliminary diagnostic approach where ultrasonographic features serve as the primary determinant for biopsy referral. Specifically, lymph nodes showing suspicious or malignancy-suspected US features should prompt tissue sampling, while isolated clinical factors such as size ≥2 cm or multiplicity, in the absence of abnormal US findings, may be managed conservatively with close follow-up. This approach may help reduce unnecessary invasive procedures while ensuring timely identification of malignant cases. Future multicenter, prospective studies are needed to validate and refine this algorithm for broader clinical use.

### Strengths and limitations

One of the main strengths of this study is the large sample size. The actual number of patients included (*n* = 500) exceeded the minimum required according to *a priori* power analysis (288 patients), thereby providing adequate statistical power for the primary comparisons and enhancing the robustness of the results. In addition, the study comprehensively evaluated multiple clinical, ultrasonographic, and pathological parameters, and representative histopathological images were included to illustrate the diagnostic spectrum. The use of multivariable logistic regression further strengthened the analysis by identifying independent predictors of malignancy.

Nonetheless, several limitations should be acknowledged. First, the retrospective and single-center design introduces the possibility of referral and selection biases, and the results may not be fully generalizable to different populations. Second, although the sample size was large overall, some subgroups (e.g., solitary lymph node cases) were relatively small, which reduced statistical power for those specific comparisons. In particular, the small number of malignancy-suspected cases led to unstable regression estimates. To address this limitation, we also performed a binary analysis combining suspicious and malignancy-suspected categories, which yielded consistent results (Supplementary Table 1). Third, ultrasound examinations were performed without elastography, which may limit generalizability to centers routinely using this technique. Finally, potential long-term outcomes could not be evaluated due to the retrospective nature of the study.

Taken together, while these limitations should be considered when interpreting the results, the strengths of the study—particularly the large cohort size, systematic evaluation of diagnostic parameters, and robust statistical analysis—enhance the credibility of the findings.

## Conclusion

While most pediatric lymphadenopathy is benign, malignancy risk clusters in select regions and larger nodes. Yet, ultrasound features—not size or site—were the only independent predictors, with suspicious patterns indicating markedly increased odds of malignancy. Embedding ultrasound-driven criteria into diagnostic pathways may minimize unnecessary biopsies and expedite timely therapy.

## Data Availability

The raw data supporting the conclusions of this article will be made available by the authors, without undue reservation.
