# Supplementary material for: Childhood lymphadenopathy: ultrasonographic predictors of malignancy in a retrospective cohort of 500 patients
Source: Front Pediatr. 2025 Nov 6;13:1663515. doi: 10.3389/fped.2025.1663515 (PMC12631647; doi:10.3389/fped.2025.1663515)
Supplement: Supplementary file 1 [file Table1.docx]

**Supplementary Table 1. Multivariable logistic regression analysis with binary ultrasound variable (Reactive vs Suspicious/Malignant)**

| **Variable** | **Adjusted OR** | **95% CI** | **p-value** |
| --- | --- | --- | --- |
| Enlarged lymph node region | 1.40 | 0.96–2.05 | 0.084 |
| Lymph node size ≥2 cm | 1.27 | 0.28–5.65 | 0.757 |
| Suspicious/Malignant vs Reactive | 55.6 | 14.3–200 | <0.001 |

Model fit: Omnibus χ² = 138.1, df = 3, p < 0.001; Nagelkerke R² = 0.674; Hosmer–Lemeshow χ² = 0.988, p = 0.320; Overall accuracy = 97.2% (specificity 99.6%, sensitivity 58.6%).
